# Evaluating the role of intern pharmacists in pharmaceutical care in hospitals in Uganda

**DOI:** 10.1080/20523211.2024.2320282

**Published:** 2024-03-11

**Authors:** Agaba Robert, Ndinawe Johnmark, Kabera Makuza Radiana, Kiiza Haulat, Segawa Ivan, Kalidi Rajab, Keren Ebong

**Affiliations:** a Pharmaceutical Society of Uganda, Pharmacum Care Uganda Project, Kampala, Uganda; b Epidemiology Unit, School of Medicine, College of Health Sciences, Makerere University, Kampala, Uganda; c Department of Pharmacy, Makerere University, Kampala, Uganda; d Department of Pharmacy, Mulago National Referral Hospital, Kampala, Uganda

**Keywords:** Pharmaceutical care practices, intern pharmacists, Interprofessional collaboration, Pharmacum Care Uganda‌, Patient care

## Abstract

**Background:**

In Uganda, there is limited adoption of pharmaceutical care in hospitals due to pharmacist shortages and limited collaboration among healthcare professionals. Intern pharmacists are deployed annually to assist in patient care to address pharmacist shortages.

**Objectives:**

Evaluate intern pharmacist’s extent of involvement in pharmaceutical care activities, assess facilitators and barriers, and explore healthcare professionals’ perceptions, attitudes, and opinions on implementation of pharmaceutical care.

**Methods:**

A mixed-method concurrent study was carried out for four months. We conducted an online survey among 107 intern pharmacists from 26 hospitals, including National Referral, Regional Referral, Private Not-For-Profit, and General hospitals; predictors of extent of involvement were identified using linear regression models, using STATA 14. 24 key informants (nurses, prescribers, pharmacists) from five hospitals were interviewed; themes were developed using a deductive thematic analysis approach.

**Results:**

Intern pharmacists had a median (Interquartile range[IQR]) age of 25 years (25-27) and 74.7% were male. About half (54.1%) of key informants were female and had a median (IQR) of 10 years (4.0-15.5) of professional experience. Interns focused on patient counseling, lacked documentation, and showed positive attitudes, and knowledge. Key informants supported implementation, but prescribers expressed dissatisfaction with interns’ performance.

**Conclusion:**

Enhancing pharmacy internship and training through developing policies and guidelines on pharmaceutical care practice, improving working conditions, and mentorship can address gaps impeding pharmaceutical care implementation by intern pharmacists.

## Introduction

Pharmaceutical care practice **(**PCP) is a pharmacist-initiated patient care while working with other healthcare professionals to address all drug-related needs, and to empower patients to take charge of their medication needs to improve patient quality of life (Morillo-verdugo and Calleja-hernández 2019). Through PCP, pharmacists and other healthcare professionals provide responsible, high-quality, and safe medical care, which is the primary goal of health systems (Sreelalitha et al. 2012). This is not commonly practiced in Uganda's healthcare system, which could be due to shortages of pharmacists in these facilities and lack of participation in inter-collaborative environments in the healthcare system, among other reasons (Obua et al. 2017). Annually, intern pharmacists are deployed by the Ministry of Health for internship which helps to address the shortage of pharmacists in hospitals.

Intern pharmacists are graduates from pharmacy schools in Uganda and from abroad who pass the pre-internship exam regulated by the Pharmaceutical Society of Uganda (PSU). They undergo a one-year paid internship which is a requirement for registration as a pharmacist in Uganda. PSU issues a temporary certificate of practice to interns who practice in hospital sites under supervision. These intern pharmacists are relied upon as a major part of the healthcare workforce in hospitals in Uganda (Bohan et al. 2020).

Although intern pharmacists are deployed in health facilities and partly address the shortage of pharmacists, their impact in hospitals is minimal because they are limited to roles such as dispensing and inventory management which can be done by pharmacy technicians. Furthermore, training in isolation during pharmacy internship negatively affects training outcomes. Therefore, interns’ engagement in direct patient care on the wards to offer pharmaceutical care services is minimal, to the disadvantage of patients who would benefit from their knowledge (Doloresco and Vermeulen 2009).

This results in patients being exposed to medication-related problems such as inappropriate therapy, drug interactions, polypharmacy, receiving drugs that are contraindicated in their disease states, and missed doses. Globally, there is a high prevalence of medication errors, and inappropriate prescribing is a major issue within healthcare systems and can often contribute to adverse drug events, many of which are preventable. This presents an enormous opportunity for pharmacists to have a significant impact on reducing these medication errors, as they have the expertise to detect, resolve, and prevent medication errors and medication-related problems, which will in turn reduce health expenditures (College of Family Physicians of Canada and Canadian Pharmacists Association 2019; Basak and Sathyanarayana 2011).

For this purpose, we have developed the Pharmacum Care Uganda project which aims to enhance the impact of intern pharmacists on patient care and is intended for implementation in hospitals in Uganda.

The Pharmacum Care Uganda project initiative is crucial, as intern pharmacists constitute the primary workforce delivering pharmacy services in Ugandan hospitals, forming the fundamental basis for achieving project objectives. This study is Phase 0 of the project which involves stakeholders namely; the Pharmaceutical Society of Uganda, healthcare professionals from internship sites, and deployed interns. It will be followed by Phase 1 (initiation phase) which will involve assessing the impact of the implementation of pharmaceutical care services in hospitals in Uganda. The project will proceed to Phase 2 (standardising phase) and Phase 3 (sustenance phase) in the consecutive proceeding years with new lots of interns respectively. The project shall establish accountability frameworks and ensure the sustainability of pharmaceutical care practices by interns in hospitals in Uganda.

This project is the first of its kind to be conducted in Uganda, focusing on intern pharmacists’ role in patient care via pharmaceutical services. Such projects have been advocated for by organisations such as the International Pharmaceutical Federation (FIP) in association with their partners and stakeholders worldwide to ensure that patients receive safe and high-quality care (Bader et al. 2019). The Common Wealth Pharmacists Association (CPA) has also advocated for such measures, which this project is undertaking to consider the role of a pharmacist in healthcare delivery and particularly their contribution to achieving sustainable development goal 3 (Rutter et al. 2018).

Therefore the aim of the study was to obtain baseline data for the planning and execution of the Pharmacumcare Uganda project in the later phases. The objectives of the study were to determine the extent of involvement of intern pharmacists in activities concerning pharmaceutical care during internship, assess for facilitators/barriers to intern pharmacists’ practice of pharmaceutical care, and explore perceptions, opinions, and attitudes of nurses, prescribers, and pharmacists towards the implementation of pharmaceutical care by intern pharmacists.

## Methods

### Study design

A concurrent mixed-methods study was conducted between 23rd July 2021 and 19th June 2022. An online cross-sectional survey collected quantitative responses using a four-point Likert scale (ranging from ‘not at all’ to ‘always’) to assess the extent of involvement, five-point Likert scales (ranging from ‘strongly agree’ to ‘strongly disagree’) for attitudes and working conditions, and a scale from (‘very poor’ to ‘excellent’) for skills assessment. The study tool for the quantitative study was uploaded on the Zoho survey portal and it had three sections i.e. Demographics, assessment of the extent of involvement in PC activities, and assessment of facilitators and barriers to PC practice. For qualitative data, we used an exploratory approach to understand the perceptions, attitudes, and opinions of hospital staff and intern supervisors (nurses, physicians, and pharmacists) towards intern pharmacists’ provision of pharmaceutical care. The study tool for key informants had three sections i.e. Demographics, perceptions, and opinion questions.

A quantitative pilot study involving 18 intern pharmacists evaluated an online questionnaire's layout and question flow for validity, seeking feedback on question significance and identifying potential removals for brevity. The reliability of responses was assessed using Cronbach's alpha. Additionally, a qualitative pilot study with 4 health professionals validated an interview guide. For content validity, we received input from independent experts for improvements in question flow and approach.

### Study setting and population

Intern pharmacists were deployed in 26 hospitals across the different regions in Uganda. The Ministry of Health mandated that all interns complete an internship for one year after graduating from university, which is a requirement for professional registration. Internship placement sites included National Referral Hospitals, Regional Referral Hospitals, General Hospitals, and Private-Not-For-Profit (PNFP) Hospitals.

Intern pharmacists deployed at the 26 internship hospitals at the time of data collection, who had access to the internet via mobile phone or computer and consented to participate were eligible to participate in the study. Healthcare professionals were purposively selected for the qualitative study from five of the 26 internship sites. These five sites were selected based on where the majority of intern pharmacists were deployed and ease of accessibility while ensuring uniform representation from all hospital categories. We selected healthcare professionals from these internship sites who consented, were available in person at the hospital during the time of data collection, and directly interacted with intern pharmacists. Healthcare professionals include pharmacists, physicians, and nurses who supervise interns, coordinate their rotations and training, or participate in policymaking at the facility.

### Inclusion and Exclusion criteria

The Uganda Medical Internship Committee deployed Intern pharmacists for their 12-month internship programme. An intern pharmacist who was invited and consented was eligible to participate in the study. Internship supervisors who interact directly with the intern pharmacists and consented to participate were eligible for the study. Pharmacists supervising intern pharmacists’ activities, Prescribers and Nurses who supervise activities in daily ward rounds, Intern coordinators at the facility, health care professionals who coordinate inter-professional pieces of training and CPDs at the facility, and health care professionals involved in policymaking at the facility.

Intern pharmacists and key informants who did not consent were excluded from the study.

### Sample size and procedures

The number of participants in our study was determined using a formula called Kish-Leslie (Singh and Masuku 2014) for estimating sample size in finite populations. We aimed for a 95% confidence level, assumed a 50% population attribute to maximise the sample size, and aimed for a 5% precision level. Initially, we arrived at a sample size of 385 participants. However, we adjusted this to 116 participants based on the total number of interns (166) in the database after the pilot study (184 interns initially minus 18 for the pilot study). From these 116 participants, we proportionately selected interns from different hospital categories: National Referral Hospitals (31/116), Regional Referrals (50/116), PNFP hospitals (20/116), and General hospitals (15/116). We selected interns from each hospital category using random numbers generated in Microsoft Excel 13 (Microsoft Corporation, Redmond, Washington, USA). With random numbers arranged in descending order (highest numerical value on top), we selected the participants corresponding to the top numbers required for each hospital category to avoid bias.

Selected participants were then contacted (and followed up via telephone) and sent a link to the online survey via email, WhatsApp, or short message service, depending on their preference. The survey questionnaire was designed in the Zoho survey (Zoho Corporation, Chennai, India). Survey responses were collated in Google Sheets (Google LLC, Mountain View, California, USA), downloaded into Microsoft Excel for cleaning, and later exported to STATA 14 (StataCorp, College Station, TX, USA) for analysis.

We also aimed to conduct a maximum of 36 interviews with 12 nurses, 12 physicians, and 12 pharmacists (3 from each of the 4 hospital categories), depending on when saturation was reached. Healthcare professionals were purposively selected and contacted to schedule the face-to-face interviews at the hospitals. Three of the authors, who were registered pharmacists, conducted semi-structured interviews using pretested interview guides (with probes), audio recorders, and additional field notes were recorded. Each interview lasted approximately 30 min.

Each data collector (three in total) was allocated one cadre profession for purposes of homogeneity and was required to do a memo every time a set of data was collected from the interviewee to establish the saturation point. Saturation for pharmacists was achieved at the 8th interview, for nurses saturation was achieved at the 7th interview, and saturation for physicians was achieved at the 9th interview. Making a total of 24 interviews from a sample space of 36 key informants.

### Statistical analysis

The primary outcome of interest was the extent of involvement in pharmaceutical care, in which responses ranged from: not at all, rarely, sometimes, and always. These categorical responses were summarised as percentages and coded from zero (not at all) to three (always) to determine the mean (with standard deviation [SD]) response. The intern pharmacist’s extent of involvement was generally considered good when the mean response (and standard deviation) was above 1.5 and poor if below. We also explored respondents’ attitudes towards pharmaceutical care, knowledge, professional skills, working conditions, and perceptions using Likert-scale questions. Responses ranged from strongly disagree to strongly agree (coded from 1 to 5) for attitudes, working conditions, and perceptions, from very poor to excellent (coded from 1 to 5) for skills, and from correct or wrong (coded 1 and 0) for knowledge questions. Internal consistency for the domains was determined using Cronbach’s alpha.

In descriptive analyses, age and working hours were summarised using median (with interquartile range [IQR]), and categorical variables such as gender and deployment site were summarised using percentages (proportions) as appropriate. For each participant, we determined their average score for each of the domains of questions (extent of involvement, attitudes, knowledge, skills, and working conditions and perceptions) and rescaled their averages to one (minimum score was 0 and maximum 1). Average scores for attitudes, knowledge, skills, working conditions, and perceptions were then compared along with demographic characteristics to the average scores for the extent of involvement in pharmaceutical care.

Linear regression was used to determine factors associated with the extent of involvement using standard statistical techniques. First, we tested for regression assumptions of the existence of a linear relation, linearity, independence, normality of residuals, equal variance, collinearity (variance inflation factor <10), and outliers. Skewed numerical variables were log (age and hours at the site) or arcsine (extent, attitudes, and skills) transformed. We then performed bivariate analysis, after which all variables were considered for multivariable analysis. Variables were then selected using a backward stepwise regression analysis, and the resultant models were checked for interaction (using the likelihood ratio test) and confounding (cut-off <10%). Final models reported standardised coefficients (Beta) as the measure of association and included significant variables and their confounders. Statistical significance was accepted at *p* < 0.05.

Pharmaceutical care implementation by intern pharmacists depends on facilitators and barriers as independent variables. The impact of these factors, along with intervening variables such as the perception and attitudes of other health workers, determines the extent of implementation(dependent variable)

### Qualitative analysis

A deductive approach to thematic analysis was used. *Apriori*-determined themes included; Inter-professional Collaboration, Attitudes of interns Towards Pharmaceutical Care, and Extent of Involvement in Pharmaceutical Care Activities, Competency, and Attitudes of key informants toward Pharmaceutical Care Implementation. Anonymization of key respondent excerpts was performed using healthcare cadres and the order of interviewing (e.g. Pharmacist 01). Audio-recorded interviews were then transcribed, coded and thematically analysed. Transcription of interviews, editing of field notes, organising and cleaning of data, and coding were performed manually by the study team. Codes were generated from excerpts from the interview scripts, and related codes were collated under *apriori*-determined themes. The analysed data were validated by contacting key informants for confirmation. Triangulation of qualitative and quantitative findings was performed for the interpretation of results.

To counter confirmation bias in thematic analysis, a collaborative approach was employed, involving multiple individuals in the independent coding of data. Coding sheets were exchanged, and discussions were held to collectively assess and refine individual judgments.

## Results

Out of 116 intern pharmacists, 107 completed the questionnaire (92.2% response rate). Overall, the interns had a median (IQR) age of 25 (25-27) years (Table 1). Of the 107 respondents, 50 (46.7%) had their internship at Regional Referral Hospitals, 80 (74.7%) were male, 54 (50.5%) were from Kampala International University and spent a median (IQR) of 45 (35-56) hours per week at the internship site. Twenty-five (23.4%) of the interns had part-time jobs and were employed for a median (IQR) of 21 (10-28) hours per week.

**Table 1.** Demographic characteristics of the intern pharmacists.

Characteristic	Response n (%) or median (IQR)
**Internship site** National Referral Hospitals	28 (26.2)
Regional Referral Hospitals	50 (46.7)
Private Not-For-Profit Hospitals	15 (14.0)
General Hospitals	14 (13.1)
**Gender** Male	80 (74.7)
Female	27 (25.3)
**Age**, years	25 (25-27)
**University Attended** Kampala International University	54 (50.5)
Makerere University	27 (25.2)
Mbarara University of Science &Technology	22 (20.6)
Other (Outside Uganda)	4 (3.7)
**Hours spent at site per week**	45 (35-56)
**Part-time job** Yes	25 (23.4)
No	82 (76.6)
**Hours spent at part-time job per week** (*N* = 25)	21 (10-28)

**Figure 1.**
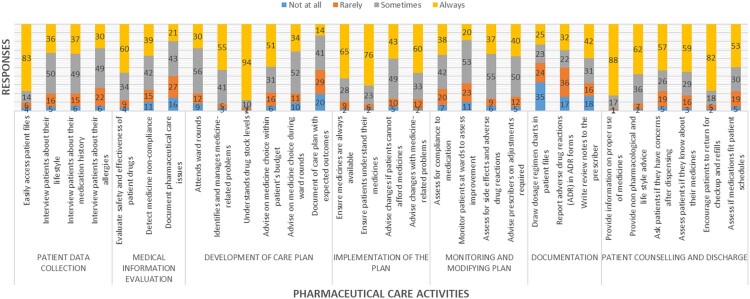
Extent of involvement in pharmaceutical care activities.

Intern pharmacists had a good extent of involvement in pharmaceutical care activities, such as accessing patient files, evaluating patient medication, identifying medicine-related problems, understanding drug-stock levels, ensuring medicine availability, patients understanding their medicines, and making changes with medicine-related problems (mean and SD > 1.5) (Figure 1). Additionally, they were greatly involved in providing information on the proper use of medicines, advising on non-pharmacological and lifestyle choices, assessing whether patients knew their medicines, and encouraging patients to return for refills and follow-up check-ups (mean and SD > 1.5). Overall, the assessment showed that interns were more involved in inpatient counseling and discharge and least involved in documentation. Potential barriers and facilitators of involvement in pharmaceutical care, such as attitudes, knowledge, skills, and working conditions, are summarised in supplementary tables (Supplementary Table S1-S4).

In bivariate analysis, attitudes (crude standardised coefficient [Beta] 0.21, *p* = 0.03), skills (crude Beta 0.47, *p* < 0.01), and working conditions (crude Beta 0.35, *p* < 0.01) were significantly associated with the extent of involvement in pharmaceutical care (Table 2). In the multivariable analysis, skills (adjusted beta 0.37, *p* < 0.01) and working conditions (adjusted beta 0.27, *p* < 0.01) were significantly associated with the extent of involvement in pharmaceutical care.

**Table 2.** Factors associated with extent of involvement in pharmaceutical care.

Factor	Crude Beta	*p*-value	Adjusted Beta	*p*-value
Attitudes*	0.21	0.03	0.05	0.59
Knowledge	0.17	0.08	–	–
Skills*	0.47	<0.01	0.37	<0.01
Work conditions/perceptions	0.35	<0.01	0.27	<0.01
Internship site				
National Referral Hospitals	1	0.74	1	0.93
Regional Referral Hospitals	−0.04	0.76	0.01	0.68
PNFP	0.03	0.84	0.04	0.57
General Hospitals	−0.02		−0.06	
Gender				
Male	1	0.45	1	0.58
Female	−0.07		−0.05	
Age (years)^¥^	0.13	0.18	–	–
University Attended				
Makerere University	1	0.10	1	0.33
Mbarara University of Science & Technology	−0.19	0.36	−0.11	0.55
Kampala International University	0.11	0.14	0.07	0.40
Other	0.15		0.08	
Hours at the internship site	0.09	0.36	–	–
Part-time job				
No	1	0.63	1	0.22
Yes	0.05		0.12	

*Arcsine-transformed ^¥^Log-transformed.

Twenty-four key informants were interviewed, eight (33.3%) of whom were from China Friendship Hospital Naguru, thirteen (54.1%) were female, nine (37.5%) were prescribers, and had an overall median (IQR) age of 37.0 (34.5-41.5) years and 10.0 (4.0-15.5) years of experience (Supplementary Table S5).

### Theme 1: Inter-professional Collaboration

Many of the prescribers reported that the intern pharmacists did not actively engage with the medical team while attending to patients.

… and rarely do they consult with the medical team. In the past (when I was an intern in 2012-2013), fellow pharmacy interns would be on the ward rounds. However, these days, I never see them during the ward round. (Prescriber 09)

Nurses, however, indicated that they were working hand in hand with the intern pharmacists.

I have to go to the pharmacist to collect the drugs; therefore, we have that professional connection. They make our lives easier and without them, it becomes hard to get the drugs. (Nurse 04)

### Theme 2: Attitudes of interns towards pharmaceutical care

Pharmacists stated that the interns had a good attitude and ensured that the right medication and doses were given to the right patients. However, pharmacists noted that interns could improve patient care but were limited by the structures in place.

… they are willing to create some change in the system but it looks like the system is not yet ready to embrace them. (Pharmacist 04)

Prescribers reported interns to have little interest in activities concerned with attending to patients directly.

They don’t seem interested and they are just trying to finish the internship and go away … I would assume that pharmaceutical care, in this case, we are talking about things like patient care and I would expect them to be involved in it … I rarely see them. (Prescriber 07)

### Theme 3: Extent of involvement in pharmaceutical care activities

Interns were more actively involved in activities that included the validation of prescriptions and checking for interactions that improved prescribing patterns.

They mainly interact with patients at the point of dispensing … this has helped to improve or to check some errors in prescriptions and guide patients on how to take their medicines. (Prescriber 03)

### Theme 4: Competency

Although interns were knowledgeable about different medications, prescribers believed that there was a need to merge this information with the patient's conditions.

… Well, I should be frank about this. Their knowledge of pharmacology is sufficient but has no linkage with the clinical aspect … the linkage is not very strong and that is why you sometimes find that for some drugs that have been repurposed, they bounce the prescriptions because they know one angle about the drug … . (Prescriber 04)

### Theme 5: Attitudes of key informants towards pharmaceutical care implementation

The medical practitioners expressed their interest in collaborating closely with the intern pharmacists to create and execute patient care plans.

The doctor may have written a drug, but if you as a pharmacist feel that drug may not be secure or to your information, you may see is not fit for the patient you have a right to advise the doctor because you may know the drug more than the doctor. (Prescriber 02)

Nurses emphasised the role of intern pharmacists to pass on key information about side effects to patients; otherwise, it would affect compliance with their medication.

A pharmacist knows more about drugs than any other person in the hospital. If you are dispensing, it is okay to tell a patient that it may increase urine output … as a patient, if I go home and experience constant urination that I did not have earlier, it would not worry me. However, if you don’t bother to give me that information, then no one will. (Nurse 04)

## Across methodological triangulation approach

Quantitative data obtained from interns satisfied objectives one and two of the study while qualitative data obtained from key informants satisfied objective three and was used to validate the credibility of quantitative data obtained from intern pharmacists. Themes previously constructed in qualitative analysis were matched with the collected interns’ data to eliminate bias and have conclusive results.

The findings revealed several areas of agreement between both datasets for example Interns were found to be more engaged in inpatient counseling and discharge procedures but less involved in documentation. This was reinforced by key informant interviews that highlighted interns’ active participation in prescription validation and checking at the dispensing point.

Both datasets identified knowledge, and skills as facilitators for pharmaceutical care practice. However, key informants emphasised the necessity for training to effectively integrate knowledge into practical application.

Working conditions were identified as barriers to pharmaceutical care practice. Quantitative data identified the following barriers in working conditions: lack of documentation materials, heavy workload at deployment sites, inadequate private space for patient interactions, absence of standard pharmaceutical care practice guidelines, and insufficient administrative support for pharmaceutical care. Key informants collaborated on these findings, asserting that the system was not yet prepared to accommodate intern pharmacists’ practice of pharmaceutical care in hospitals.

Contradictory findings in both data sets included; Interns reported to have good attitudes towards pharmaceutical care while prescribers indicated that their attitude was poor concerning activities related to in-ward patient care.

## Discussion

Intern pharmacists were primarily involved in dispensing medicines and counseling patients at the time of discharge, which correlated with their professional skills, working conditions, and gaps in inter-professional collaboration among the healthcare team. It is important to note that intern pharmacist supervisors had a positive attitude towards pharmaceutical care implementation, despite interns’ less involvement in direct patient care (Oteba et al. 2018).

According to a study conducted in Nigeria, over 90% of pharmacists were primarily involved in counseling patients to ensure adherence and providing information on the use of medicines at the hospital (Adisa and Anifowose 2019). In Pakistan, pharmacists agreed that their curriculum was focused on dispensing and manufacturing, which influenced their activities, but they were willing to engage in pharmaceutical care. They also believed that participating in clinical rounds and continuous medical education activities with other healthcare professionals would enable pharmacists to be recognised as clinical members of the multidisciplinary team (Khan et al. 2020).

The study found documentation to be the least performed pharmaceutical care activity by intern pharmacists. However, the American Society of Hospital Pharmacists emphasises the importance of documentation in ensuring continuity of patient management, accountability, and value of the pharmacist who attended to the patient (American Society of Hospital Pharmacists 2003). To address this issue, it would be necessary to adopt a standard documentation format for pharmacists to write their pharmaceutical care plans in the patient file. This would enhance patients’ follow-up and monitoring as they move from one level and state of care to another (Onyebuchi et al. 2015).

The study found that intern pharmacists were knowledgeable in pharmaceutical care, but their working conditions limited them to dispensing medications with less involvement in direct patient care. This suggests a need to extend their scope of practice to improve patient outcomes (Chiutsi et al. 2022). However, in most hospital facilities, there are no existing policies and structures that allow intern pharmacists and pharmacists to practice pharmaceutical care, hence being confined to dispensaries and the role of supply chain management (Khan et al. 2020). The pharmacist undergraduate training curriculum is still centered on traditional roles and would require it to be altered to become more patient-centered to equip pharmacists to make recommendations using a patient-oriented approach (Sakeena et al. 2019). This would improve their clinical skills and confidence while in pharmacy school, thereby using these skills to boost pharmaceutical care practices (Al-Quteimat and Amer 2016).

In the study, prescribers reported intern pharmacists to have poor attitudes toward patient care (AbuRuz et al. 2012), and some studies also reported doctors to be uncomfortable with pharmacists attending to patients (Azhar et al. 2010). However, prescribers expressed a positive attitude towards implementing pharmaceutical care and agreed that intern pharmacists should provide pharmaceutical care to patients in hospitals as one of their core roles. A recent study in Tehran suggested that increasing the awareness of physicians about the expanded role of pharmacists in multidisciplinary teams could resolve this issue (Alipour et al. 2018).

In the study, it was found that while intern pharmacists were deemed knowledgeable in pharmaceutical care by prescribers, there was a need for them to apply this knowledge more directly to patient conditions. This includes roles such as monitoring the efficacy of pharmacotherapy in patients and documenting adverse drug reactions (Bohan et al. 2020). In Australia, the involvement of pharmacists in tasks such as medication chart reviews, ward rounds, and therapeutic drug monitoring was found to be beneficial in improving patient outcomes (Natalia Krzyżaniak and Bajorek 2019). However, the absence of policies and standard guidelines on pharmaceutical care practice during internships has resulted in its limited popularity among other healthcare professionals (Agaceta et al. 2014). The implementation of these guidelines, on the other hand, has been shown to increase the acceptance of pharmacists’ recommendations during clinical rounds (Mekonnen et al. 2018).

In conclusion, the government must establish comprehensive guidelines and policies related to pharmaceutical care for the successful implementation of pharmaceutical care practice in hospitals to improve the quality of patient care.

### Strengths and limitations of the study

To our knowledge, this study is the first to be conducted in Uganda to assess the factors that affect the practice of pharmaceutical care by intern pharmacists. The mixed method approach from two study participants, i.e. intern pharmacists and key informants (supervisors), enabled us to compare and confirm the actual factors.

The research uncovered barriers at the internship sites that hinder pharmaceutical care practice by interns and deepened insights into inter professional relationships informing plans for interprofessional education and collaboration. Additionally, the identified facilitators of pharmaceutical care practice will be leveraged to reinforce and enhance pharmaceutical care services as part of the project's later phases.

The study will lay the groundwork to improve pharmacy education curricula in universities and propose the development of a nationwide plan for pharmaceutical care implementation, aiming to extend these efforts to pharmacy students, intern pharmacists, and registered pharmacists. Additionally, the study offered insights into the working environment of intern pharmacists. These will facilitate the development of pharmaceutical care guidelines and the efficient integration of their roles within the healthcare teams to cretae a collaborative environment during hospital internships.

A potential limitation is the self-reporting of competencies by intern pharmacists, which did not reflect the respondent’s actual competence. Another potential limitation was that the key informants may have provided us with socially desirable responses. A further limitation is that the information of key informants from the selected hospitals may not apply to other hospitals.

## Conclusion

Intern pharmacists were knowledgeable and willing to practice pharmaceutical care, although they were inclined to dispense and seldom documented the activities. Much more effort should be put into overcoming barriers and strengthening facilitators to the practice of pharmaceutical care in hospitals. Further engagement with stakeholders would be necessary to recruit more pharmacists, who would supervise and mentor interns during their ward rotations, to improve their interprofessional skills within the healthcare team. Furthermore, revising the curriculum of pharmacists’ undergraduate training, and introducing guidelines and policies on pharmaceutical care would enable intern pharmacists to transition from their traditional roles to taking on more direct-patient care roles. Further research should be conducted to investigate; professional relationships among different health professional cadres in both public and private facilities in Uganda, medicine use problems, and their impact on the health of Ugandan citizens.

## Supplementary Material

Supplemental Material Table_S5_KII_Demographics

Supplemental Material Table_S4_Working_Conditions

Supplemental Material Table_S3_Skills

Supplemental Material Table_S2_Knowledge

Supplemental Material Table_S1_Attitude

## Data Availability

The datasets generated during and/or analysed during the study have been stored on the Pharmacum Care Uganda Google Drive and thematic analysis sheets in physical folder. All are only available from the corresponding author upon request. The study documents were securely kept, and the de-identified database was password-protected.
