# Supplementary material for: Evaluating the role of intern pharmacists in pharmaceutical care in hospitals in Uganda
Source: J Pharm Policy Pract. 2024 Mar 11;17(1):2320282. doi: 10.1080/20523211.2024.2320282 (PMC10930095; doi:10.1080/20523211.2024.2320282)
Supplement: Supplemental Material Table_S5_KII_Demographics [file JPPP_A_2320282_SM6154.pdf]

Supplementary Table S5: List of Key Informants

Study ID	Internship Site	Gender	Designation	Age (years)	Experience (years)
Pharmacist 01	Nsambya Hospital	Female	Pharmacist	32	4
Pharmacist 02	Nsambya Hospital	Female	Pharmacist	34	6
Pharmacist 03	Naguru Hospital	Male	Pharmacist	26	1
Nurse 01	Nsambya Hospital	Female	Nurse	39	15
Nurse 02	Nsambya Hospital	Female	Nurse	42	16
Pharmacist 04	Naguru Hospital	Male	Pharmacist	34	12
Prescriber 01	Naguru Hospital	Male	Prescriber	41	4
Pharmacist 05	Naguru Hospital	Female	Pharmacist	48	4
Nurse 03	Naguru Hospital	Female	Nurse	35	2
Prescriber 02	Naguru Hospital	Male	Prescriber	29	2
Prescriber 03	Naguru Hospital	Male	Prescriber	36	10
Prescriber 04	Nsambya Hospital	Male	Prescriber	51	13
Nurse 04	Naguru Hospital	Female	Nurse	38	5
Prescriber 05	Lira Hospital	Male	Prescriber	35	10
Prescriber 06	Lira Hospital	Male	Prescriber	37	10
Prescriber 07	Nsambya Hospital	Female	Prescriber	40	16
Prescriber 08	Mulago Hospital	Female	Prescriber	37	8
Pharmacist 06	Mulago Hospital	Female	Pharmacist	55	16
Nurse 05	Mulago Hospital	Female	Nurse	40	19
Nurse 06	Kirudu Hospital	Female	Nurse	48	20
Pharmacist 07	Kirudu Hospital	Male	Pharmacist	35	3
Nurse 07	Kirudu Hospital	Female	Nurse	52	30
Pharmacist 08	Lira Hospital	Male	Pharmacist	36	5
Prescriber 09	Mulago Hospital	Male	Prescriber	34	11
