# Supplementary material for: Evaluating the role of intern pharmacists in pharmaceutical care in hospitals in Uganda
Source: J Pharm Policy Pract. 2024 Mar 11;17(1):2320282. doi: 10.1080/20523211.2024.2320282 (PMC10930095; doi:10.1080/20523211.2024.2320282)
Supplement: Supplemental Material Table_S4_Working_Conditions [file JPPP_A_2320282_SM6149.pdf]

**Supplementary Table S4: Working Conditions at the Internship Site**

| | Strongly disagree (1), n (%) | Disagree (2), n (%) | Not sure (3), n (%) | Agree (4), n (%) | Strongly Agree (5), n (%) | Mean (SD) |
|---|---|---|---|---|---|---|
| **Resources (Cronbach α = 0.7)** | | | | | | |
| Undergraduate PC training curriculum is sufficient | 4 (3.8) | 47 (44.3) | 8 (7.6) | 37 (34.9) | 10 (9.4) | 3.0 (1.2) |
| I have enough time to perform PC in this hospital | 8 (7.6) | 39 (36.8) | 39 (36.8) | 32 (30.2) | 19 (17.9) | 3.1(1.3) |
| There are documentation materials for PC | 18 (17.1) | 39 (37.1) | 7 (6.7) | 28 (26.7) | 13 (12.4) | 2.8 (1.3) |
| Hard copy references available and accessible | 11 (10.4) | 29 (27.4) | 1 (0.9) | 26 (24.5) | 39 (36.8) | 3.5 (1.5) |
| Continuous professional training is provided | 7 (6.6) | 12 (11.3) | 6 (5.7) | 50 (47.2) | 31 (29.3) | 3.8 (1.2) |
| **Motivation Factors (Cronbach α = 0.5)** | | | | | | |
| There is no heavy workload at my site of deployment | 27 (25.5) | 43 (40.6) | 6 (5.7) | 24 (22.6) | 6 (5.7) | 2.4 (1.2) |
| Internship prepares me for pre-registration exams | 3 (2.8) | 6 (5.7) | 8 (7.6) | 39 (36.8) | 50 (47.2) | 4.2 (1.0)* |
| Conditions allow me to practice PC at my will | 13 (12.4) | 32 (30.5) | 13 (12.4) | 30 (28.6) | 17 (16.2) | 3.1 (1.3) |
| **Social Influences (Cronbach α = 0.5)** | | | | | | |
| I efficiently collaborate with other professionals | 0 (0.0) | 8 (7.6) | 4 (3.8) | 60 (56.6) | 34 (32.1) | 4.1 (0.8)* |
| There's enough supervision and mentorship at wards | 13 (12.3) | 39 (36.8) | 8 (7.6) | 30 (28.3) | 16 (15.1) | 3.0 (1.3) |
| Patients are eager for PC services in my unit | 3 (2.8) | 17 (16.0) | 24 (22.6) | 45 (42.5) | 17 (16.0) | 3.5 (1.0) |
| I can easily access patients at wards | 3 (2.8) | 10 (9.4) | 6 (5.7) | 47 (44.3) | 40 (37.7) | 4.0 (1.0)* |
| **Space or Privacy** | | | | | | |
| There is a private space for attending to patients | 14 (13.3) | 39 (37.1) | 9 (8.6) | 31 (29.5) | 12 (11.4) | 2.9 (1.2) |
| **Healthcare Policy** | | | | | | |
| There are standard guidelines for PC practice | 14 (13.2) | 47 (44.3) | 17 (16.0) | 20 (18.9) | 8 (7.6) | 2.6 (1.2) |
| There's administrative support for implementing PC | 19 (17.9) | 34 (32.1) | 17 (16.0) | 29 (27.4) | 7 (6.6) | 2.7 (1.2) |

PC: Pharmaceutical care
*Good working conditions (Mean and SD>3)
