# Supplementary material for: Evaluating the role of intern pharmacists in pharmaceutical care in hospitals in Uganda
Source: J Pharm Policy Pract. 2024 Mar 11;17(1):2320282. doi: 10.1080/20523211.2024.2320282 (PMC10930095; doi:10.1080/20523211.2024.2320282)
Supplement: Supplemental Material Table_S3_Skills [file JPPP_A_2320282_SM6147.pdf]

Supplementary Table S3: Professional Skills

	Very Poor (1), n (%)	Poor (2), n (%)	Fair (3), n (%)	Good (4), n (%)	Excellent (5), n (%)	Mean (SD)
**Interpersonal Relationship Skills (Cronbach α = 0.6)**						
1. Maintenance of a climate of mutual respect and shared values with other health care cadres	0 (0.0)	0 (0.0)	9 (8.5)	43 (40.6)	54 (50.9)	4.4 (0.6)*
2. Ability to use knowledge of my role and those of other health professionals to address patients' needs	0 (0.0)	0 (0.0)	8 (7.6)	57 (53.8)	41 (38.7)	4.3 (0.6)*
3. Communicate responsively and responsibly to support teamwork	0 (0.0)	0 (0.0)	7 (6.6)	43 (40.6)	56 (52.8)	4.5 (0.6)*
4. Application of relationship-building values and team dynamics to perform effectively while attending to patients	0 (0.0)	1 (0.9)	16 (15.1)	46 (43.4)	43 (40.6)	4.2 (0.7)*
**Communication Skills (Cronbach α = 0.7)**						
1. Critical paying attention and maintaining eye contact while attending to patients	1 (0.9)	0 (0.0)	6 (5.7)	41 (38.7)	58 (54.7)	4.5 (0.7)*
2. Use of verbal, non-verbal, and written communications skills to communicate appropriately with patients.	0 (0.0)	0 (0.0)	8 (7.6)	32 (30.2)	66 (62.3)	4.5 (0.6)*
3. Ability to use all senses to evaluate patients' use of medication at the hospital to ensure adherence	0 (0.0)	0 (0.0)	21 (19.8)	48 (45.3)	37 (34.9)	4.2 (0.7)*
4. Understanding patient's feelings/ emotions and enthusiastically provide medication support	0 (0.0)	2 (1.9)	15 (14.2)	49 (46.2)	40 (37.7)	4.2 (0.7)*
**Implementation Skills (Cronbach α = 0.6)**						
1. Collection of patients' medication history data by reviewing patient files and clerking them.	1 (0.9)	5 (4.7)	25 (23.6)	51 (48.1)	24 (22.6)	3.9 (0.9)*
2. Utilize data to identify patient needs and prioritize them	0 (0.0)	0 (0.0)	14 (13.2)	58 (54.7)	34 (32.1)	4.2 (0.6)*
3. Detection of medication use problems such as drug interactions, underdose, overdose, and irrational drug use	0 (0.0)	0 (0.0)	6 (5.7)	47 (44.3)	53 (50.0)	4.4 (0.6)*
4. Devise an optimum drug regimen to meet patient needs depending on body weight, age, and sex	2 (1.9)	1 (0.9)	17 (16.0)	54 (50.9)	32 (30.2)	4.1 (0.8)*
**Therapeutic Monitoring Skills (Cronbach α = 0.8)**						

1. Ability to set target subjective and objective parameters for evaluating improvement in wellbeing	0 (0.0)	5 (4.7)	25 (23.6)	59 (55.7)	17 (16.0)	3.8 (0.7)*
2. Ability to assess subjective and objective data to determine patients' response to therapy	0 (0.0)	4 (3.8)	15 (14.2)	64 (60.4)	23 (21.7)	4.0 (0.7)*
3. Ability to detect unusual and unexpected event that's not reported about the drug in the monogram	2 (1.9)	10 (9.4)	37 (34.9)	43 (40.6)	14 (13.2)	3.5 (0.9)
4. Adjustment of doses of drugs depending on patient factors during monitoring of therapy	0 (0.0)	3 (2.8)	17 (16.0)	53 (50.0)	33 (31.1)	4.1 (0.8)*

*Good professional skills (Mean and SD>3)
