# Supplementary material for: Evaluating the role of intern pharmacists in pharmaceutical care in hospitals in Uganda
Source: J Pharm Policy Pract. 2024 Mar 11;17(1):2320282. doi: 10.1080/20523211.2024.2320282 (PMC10930095; doi:10.1080/20523211.2024.2320282)
Supplement: Supplemental Material Table_S2_Knowledge [file JPPP_A_2320282_SM6146.pdf]

Supplementary Table S2: Knowledge Assessment

	Correct, n (%)	Wrong, n (%)
1. Pharmaceutical care is responsible provision of drug therapy to achieve definite outcomes that improve the patient’s quality of life	106 (100.0)	0 (0.0)
2. Pharmaceutical care is a philosophy of practice where pharmacists work with patients only to optimize the outcomes of medication therapy	62 (58.5)	44 (41.5)
3. Medication optimization focuses on how to ensure safe and effective medicines use to obtain the best possible therapeutic outcomes	101 (95.3)	5 (4.7)
4. The primary purpose of the assessment is to determine to what extent the patients’ drug-related needs are being met	89 (84.0)	17 (16.0)
5. Having a drug regimen that is too complex is a common reason why patients are non-compliant	82 (77.4)	24 (22.6)
6. The patient’s age and sex is significantly associated with compliance with medication therapy	22 (20.8)	84 (79.2)
7. Documentation of the care provided is among the vital elements of the pharmaceutical practice process	105 (99.1)	1 (0.9)
