# Supplementary material for: Evaluating the role of intern pharmacists in pharmaceutical care in hospitals in Uganda
Source: J Pharm Policy Pract. 2024 Mar 11;17(1):2320282. doi: 10.1080/20523211.2024.2320282 (PMC10930095; doi:10.1080/20523211.2024.2320282)
Supplement: Supplemental Material Table_S1_Attitude [file JPPP_A_2320282_SM6080.pdf]

Supplementary Table S1: Attitudes towards Pharmaceutical Care

	Strongly disagree (1), n (%)	Disagree (2), n (%)	Not sure (3), n (%)	Agree (4), n (%)	Strongly Agree (5), n (%)	Mean (SD)
It is my role to provide PC services in this hospital	1 (0.9)	0 (0.0)	0 (0.0)	23 (21.7)	82 (77.4)	4.7 (0.6)*
The practice of PC is not important to be considered	86 (81.1)	15 (14.2)	0 (0.0)	1 (0.9)	4 (3.8)	4.9 (0.3)*
I feel that PC practice will improve patient health	0 (0.0)	0 (0.0)	0 (0.0)	9 (8.5)	97 (91.5)	4.7 (0.6)*
PC is the right direction for the profession to be headed	1 (0.9)	1 (0.9)	1 (0.9)	20 (18.9)	83 (78.3)	4.9 (0.3)*
Providing PC will negatively affect my ward relationships≠	39 (36.8)	35 (33.0)	14 (13.2)	13 (12.3)	5 (4.7)	1.3 (0.9)
Practicing PC will increase patients' confidence¥	0 (0.0)	0 (0.0)	1 (0.9)	10 (9.4)	95 (89.6)	2.2 (1.2)
I prefer fewer challenging tasks like dispensing	47 (44.3)	40 (37.7)	4 (3.8)	14 (13.2)	1 (0.9)	1.9 (1.0)
Providing PC does not fall in the scope of my work	71 (67.0)	26 (24.5)	5 (4.7)	1 (0.9)	3 (2.8)	1.5 (0.9)

Cronbach α = 0.5

PC: Pharmaceutical care

≠Relationships with the physicians and nurses at the ward

¥Patient's confidence in the Pharmacy profession

*Good attitude (Mean and SD>3)
